# Editorial: Rising stars in inflammation 2021

**DOI:** 10.3389/fimmu.2023.1193694

**Published:** 2023-05-03

**Authors:** A. Baragetti, J. Suurmond, P. E. Marques, L. P. Tavares

**Affiliations:** ^1^ Department of Pharmacological and Biomolecular Sciences "Rodolfo Paoletti", University of Milan, Milan, Italy; ^2^ Department of Rheumatology, Leiden University Medical Center (LUMC), Leiden, Netherlands; ^3^ Rega Institute for Medical Research, KU Leuven, Leuven, Belgium; ^4^ Pulmonary and Critical Care Medicine Division, Department of Medicine, Brigham and Women’s Hospital and Harvard Medical School, Boston, MA, United States

**Keywords:** inflammation, innate immunity, adaptive immunity, chronic pathologies, cellular reprogramming

Inflammation encompasses a wide range of cellular and molecular mechanisms that connect the pathophysiological aspects underlying most acute and chronic morbidities, such as cancers, cardiovascular, metabolic inflammatory diseases, autoimmune diseases, and neurodegeneration (1–5).

Discussing inflammation entails embracing a large spectrum of scientific backgrounds, interconnected by integrating different investigative approaches. Consequently, inflammation represents the perfect subject for scientific collaborations between early career researchers. On this shared foundation, they can explore rigorous, innovative, and cutting-edge strategies for advancing basic and translational science in the fields of biology, biotechnology, and biomedicine, among many others. Through this, they can aspire to become leaders in academia and science education.

To satisfy this sense of excitement, *Frontiers in Immunology* launched in 2021 the Research Topic “*Rising Stars in Inflammation 2021*”, aimed at encouraging young scientists who have the potential to become future leaders in inflammation research to submit their works across the entire breadth of the field of inflammation and to showcase advancements in theory, experiment, and methodology with applications to compelling aspects of inflammation.

This Research Topic, moderated by four independent young editors, has been a successful experience, with 10 articles published by international research groups, including original articles and reviews.

This Research Topic includes a heterogeneous set of topics, spanning from “technical” research manuscripts, outlining methodologies to optimize the study of immune cells, to state-of-the-art reviews on topics of inflammation and research manuscripts with a basic science or translational perspective.

Among the technical research manuscripts, Blanter et al. carefully described the optimal methods for the isolation and functional characterization of neutrophils. Through a well-conducted methodology, the authors suggested the use of the magnetic-based separation of this immune cell subset for specific functional assays. Additionally, in this Research Topic, Rumianek et al. presented an interesting transgenic reporter mouse to specifically target and label tissue-resident macrophages by using a tamoxifen-inducible Cre recombinase under the control of the human CD68 promoter (hCD68-CreERT2). The authors demonstrated that this tool guarantees the specific and long-term (6 weeks) targeting of tissue-resident macrophages with negligible labeling of other myeloid cells. This model provides an opportunity to gain more insights into the phenotype and function of different macrophage subsets in both healthy and diseased states.

Furthermore, this issue showcases different perspectives on the mechanisms by which both the innate and adaptive immune systems respond to pathogens and antigens and how immune cells “adapt” to chronic inflammatory and metabolic status in the long term. For instance, Lopes et al. described the role of Kupffer cells (KCs), the hepatic tissue-resident macrophage, in controlling systemic infection post-liver injury due to acetaminophen (APAP) overdose. More specifically, KCs, whose density in the liver decreases during the development of liver injury, rapidly repopulated the areas of necrosis to restore the liver firewall function. Hence, this study not only describes the impaired function of KCs in liver injury, but it also points to future therapeutic strategies directed to restoring liver function post-injury. On the other hand, Collier et al.‘s study showed an interesting correlation of systemic inflammatory markers and innate immune cell activity in preschool children. Of interest, the authors found that a higher body mass index positively correlates with increased circulating levels of inflammatory markers and a pro-inflammatory potential by monocytes, suggesting that this imprinting can potentially indicate a higher risk for the development of non-communicable diseases later in life.

Wan et al. reviewed in detail the inflammatory immune-associated enhancer RNAs (eRNAs), providing a comprehensive overview about their molecular biology, outlining their involvement in inflammatory immune diseases and tumor inflammation, and discussing their therapeutic applications. In addition, Xiao et al. presented a comprehensive review about another target for inflammation control. The authors reviewed the non-classic role of transient receptor potential vanilloid 1 (TRPV1) channels in controlling T-cell-mediated inflammatory responses and diseases.

Aiding the complex understanding of inflammation as a whole, two reviews described the role of cellular organelles such as mitochondria and endosomes in immune cell activity. Cao et al. assessed the current knowledge on the role of mitochondria in shaping neutrophil response and phenotype, including regulating oxidative burden, migration, and the effector mechanisms of these short-living cells in tissues. Meanwhile, Gonzales and Canton reviewed the emerging evidence for the active cytosolic transfer of diverse macromolecular “danger” signals across endocytic organelle membranes in phagocytes. This process may have important implications for the cellular response to macromolecules such as double-stranded DNA, lipopolysaccharide, and peptidoglycan and mediating inflammatory responses.

An original research article by Nakandakari-Higa et al. describes their findings on a novel humanized mouse model designed to replicate and better comprehend SARS-Cov-2 infection. The authors elegantly developed a minimally edited tool to study the maturation of B cells and immune responses to SARS-Cov2 infection.

Finally, as in other fields of research, the experimental evidence needs to find clinical translation. The large epidemiological study of Hong et al., also published in this issue, recapitulated the abovementioned studies describing the mechanisms of inflammation. In this cross-sectional survey of more than 2600 subjects, the authors found significant correlations between markers of systemic inflammation, dyslipidemia, and lower antioxidant capacity.

In conclusion, we hope that the readers appreciate the varied research included in this Research Topic, “*Rising Stars in Inflammation*”. The editorial team put substantial effort into screening and evaluating the high-quality manuscripts produced by young researchers and their groups. We hope that this issue will aid in the efforts to foster collaboration among and support to young researchers, as these are crucial elements in their progression towards more advanced stages in their scientific careers.

## Author contributions

AB, JS, LPT and PEM contributed in writing and editing the original manuscript.
